# Multigene human artificial chromosome vector delivery with herpes simplex virus 1 amplicons

**DOI:** 10.1016/j.yexcr.2020.111840

**Published:** 2020-03-15

**Authors:** David YL Chan, Daniela Moralli, Lucy Wheatley, Julia D. Jankowska, Zoia L. Monaco

**Affiliations:** a Wellcome Centre for Human Genetics, University of Oxford, Oxford, UK; b Assisted Reproductive Technology Unit, Department of Obstetrics and Gynaecology, Faculty of Medicine, The Chinese University of Hong Kong, Hong Kong; c Department of Biomedical Engineering, Tufts University, Medford, MA, USA

**Keywords:** Human artificial chromosome (HAC), Herpes simplex Virus-1 (HSV-1) amplicons, Multigene delivery, HPRT gene expression, Gene therapy

## Abstract

Gene expression studies and gene therapy require efficient gene delivery into cells. Different technologies by viral and non-viral mechanisms have been used for gene delivery into cells. Small gene vectors transfer across the cell membrane with a relatively high efficiency, but not large genes or entire loci spanning several kilobases, which do not remain intact following introduction. Previously, we developed an efficient delivery system based on herpes virus simplex type 1 (HSV-1) amplicons to transfer large fragments of DNA incorporated in human artificial chromosome (HAC) vectors into the nucleus of human cells. The HSV-1 amplicon lacks the signals for cleavage and replication of its own genome, yet each amplicon has the capacity to incorporate up to 150 kb of exogenous DNA. In this study, we investigated whether the capacity of gene delivery could be increased by simultaneously introducing multiple HSV-1 modified amplicons carrying a gene expressing HAC vector into cells with the aim of generating a single artificial chromosome containing the desired genes. Following co-transduction of two HSV-1 HAC amplicons, artificial chromosomes were successfully generated containing the introduced genes, which were appropriately expressed in different human cell types.

## 1 Introduction

Gene expression vectors are useful for monitoring expression in human cells and complementation studies of genetic disorders for gene therapy. Different types of vectors have been utilised for efficient and intact transfer across the cell membrane. Non-viral methods have included transfer of genes in conjunction with liposomes, peptides, polymers, transposases or naked DNA introduced mechanically via electroporation, microinjection, sonoporation, magnetofection and gene gun delivery. Viral vectors include retrovirus, adenovirus adeno-associated virus, lentivirus, alphavirus, baculovirus and herpes virus [1]. Most gene delivery vectors can accommodate several small genes or a single large gene as the cDNA up to 14 kb. However, regulatory sequences surrounding the gene may also be required for full expression. For larger genes, genomic clones as bacterial or yeast artificial chromosomes (BAC, YAC) were constructed to incorporate whole loci up to several hundred kilobases in size, however delivery of these larger vectors into cells was inefficient due to ectopic DNA degradation following transfer [2].

We developed a successful method to transfer large DNA intact in human artificial chromosome vectors (HAC) modified with Herpes Virus Simplex −1 (HSV-1) sequences as amplicons for delivery [3]. HAC vectors are autonomous, non-integrating vectors that incorporate larger genomic sequences. Once formed as a functional artificial chromosome within the cell, they can be detected and monitored by fluorescent in situ hybridisation (FISH) and immunoFISH. HAC amplicons efficiently form HAC in a variety of cell types including human embryonic stem cells, and genes are effectively expressed from the HAC [3,4]. The size of the insert DNA in the amplicon is 150 kb, which is the packaging capacity of the virion [5]. The HAC vector requires approximately 40 kb of alpha satellite (alphoid, α) DNA to seed the formation of a new centromere and develop a stable HAC, and the remaining 110 kb is available for incorporating therapeutic genes and their regulatory regions.

Previously, we showed that the HSV-1 amplicon HAC system is ideal for single gene delivery. However, genetic disorders resulting from possible defects in large genes, gene locus or multiple genes (polygenic) would require multigenic delivery into cells for complementation of the defect and full gene expression. Developing a single HAC vector containing more than one large gene or an extended gene loci would pose major cloning challenges in vector construction. This study investigated whether the simultaneous co-infection of two different HSV-1 amplicon containing HAC vectors, would generate a single HAC with genes appropriately expressed. Previous reports using baculovirus have shown that human cells can be simultaneously infected with multiple viruses and sustain transient multigene expression [6]. However, baculovirus has a capacity of up to 38 kb only [7].

In the initial experiment, an HSV-1 amplicon containing a HAC vector with chromosome 17α DNA, and a second HSV-1 amplicon containing 21α DNA were introduced into HT1080 cells. A functional HAC containing both 17α and 21α DNA was formed in these cells. In a further experiment, a HSV-1 amplicon containing a HAC vector with 17α DNA and a marker gene, and a second HSV-1 amplicon containing the hypoxanthine guanine phosphoribosyl transferase (HPRT) gene but lacking alphoid DNA, were co-transduced into human cells to determine if a single stable artificial chromosome would be generated containing the HPRT gene. Our results showed that a functional artificial chromosome containing 17α DNA and the HPRT gene was formed, indicating that the presence of alphoid DNA is only required in one HAC vector to seed a functional HAC following co-transduction of two amplicons. The HPRT gene was expressed, and the HPRT defect was complemented in the HT1080 deficient cells.

The major advantage of the co-transduction technique, is that additional HSV-1 amplicons containing gene HAC vectors can exploit the full size carrying capacity (150 kb) of the amplicon without the need to incorporate centromeric alphoid DNA in each HSV-1 amplicon HAC vector that is co-transduced into cells.

## 2 Materials and methods

### 2.1 Vectors

The 17 RFP vector is an approximately 140 Kb construct, comprising a BAC backbone carrying 120 kb of human α DNA from chromosome 17, the RFP gene under control of the HSV-1 immediate/early promoter, the blasticidin resistance gene under control of the pUB promoter, and the HSV-1 origin of replication (*OriS*) and packaging (*pac*) elements [8].

The 21 GFP (75 Kb) and HPRT GFP (8 Kb) vectors were described previously [3]. Briefly, both constructs contain the G418 resistance gene under the control of the SV40 promoter, the GFP gene under control of HSV-1 immediate/early promoter, the HPRT minigene, under control of the PGK gene promoter as well as the *OriS* and *pac* elements. In addition 21 GFP also contains 60 kb of alphoid DNA, from chromosome 21.

### 2.2 Cell lines

HT1080 cells (wild type WT, and HPRT-, ATCC CCL-121) were grown in Dulbecco's modified Eagle (DMEM) high glucose (Sigma Aldrich) 10% foetal bovine serum (FBS), supplemented with 1x GlutamaxTM-1 (Sigma Aldrich), penicillin 100 U/ml (Sigma Aldrich) and streptomycin 100 μg/ml (Sigma Aldrich). G16-9 [3] cells were grown in the same medium, with the addition of 200 μg/ml hygromycin (InVivoGen). The human induced pluripotent stem cell (hiPS) line DF19-9-11T.H was obtained from WiCell Research Institute (Wisconsin, USA). The human embryonic cells (hESc) HUES-2 cells were obtained from Douglas Melton (Harvard University, USA) under the license from the UK Stem Cell Steering Committee. The hiPS and hESc were grown on inactivated mouse embryo fibroblasts (iMEFs) medium composed of DMEM-F/12(Sigma Aldrich) medium supplemented with 20% (v/v) KnockOut Serum Replacement (ThermoFisher Scientific), 0.1 mM non-essential amino acids (Sigma Aldrich), 1x GlutamaxTM-1 (Sigma Aldrich), 0.1 mM β-mercaptoethanol, 10 ng/ml basic fibroblast growth factor (ThermoFisher Scientific) and 1% (v/v) penicillin and streptomycin.

### 2.3 Amplicon preparation and transduction

Amplicons were prepared and concentrated as described previously [3,4]. For dual transduction, cells were seeded into 6 well-plates and simultaneously transduced at multiplicity of infection (MOI) 1 and 5 of both amplicons types, in 1 ml of culture medium. The plate was then centrifuged at 750g for 45 min [10]. The HT1080 cells were co-transduced with 17 RFP and 21 GFP or HPRT GFP, selection was applied after 24 h with 5 μg/ml blasticidin and 120 μg/ml G418 and/or HAT 1x (Sigma Aldrich) and monitored until individual clones were observed.

### 2.4 FISH and ImmunoFISH

Metaphase spread preparation and FISH was carried out as described previously [3,4,9]. For immunoFISH, metaphase spreads fixed in Carnoy's fixative were stained with *anti*-Cenp C (Abcam ab50974) and hybridised according to Beh and colleagues [11].

### 2.5 HPRT analysis

Protein extracts were prepared using the Total Protein Extraction kit (Merck Millipore), according to the manufacturer instructions. Western blots were probed with *anti*-HRPT (Abcam ab10479) and anti GAPDH (Abcam ab9485) antibodies, detected with HRP conjugated secondary antibodies.

Total RNA was extracted using the RNeasy Mini Kit (Qiagen) following manufacturers' instructions, and reverse transcribed with the Quanta Biosciences qScript cDNA super mix system. Quantitative real time analysis of HPRT expression was conducted using Biorad Sybergreen with the following primers: HPRT F 5′-GCCCCAAAATGGTTAAGGTT-3′ HPRT R 5′-CAAGGGCATATCCAACAACA-3'; GAPDH F 5′-GAGTCAACGGATTTGGTCGT-3′ GAPDH R 5′-GACAAGCTTCCCGTTCTCAG-3. The quantitative RT-PCR data was analysed using the 2^−ΔCt^ method.

## 3 Results

**Fig. 1:**
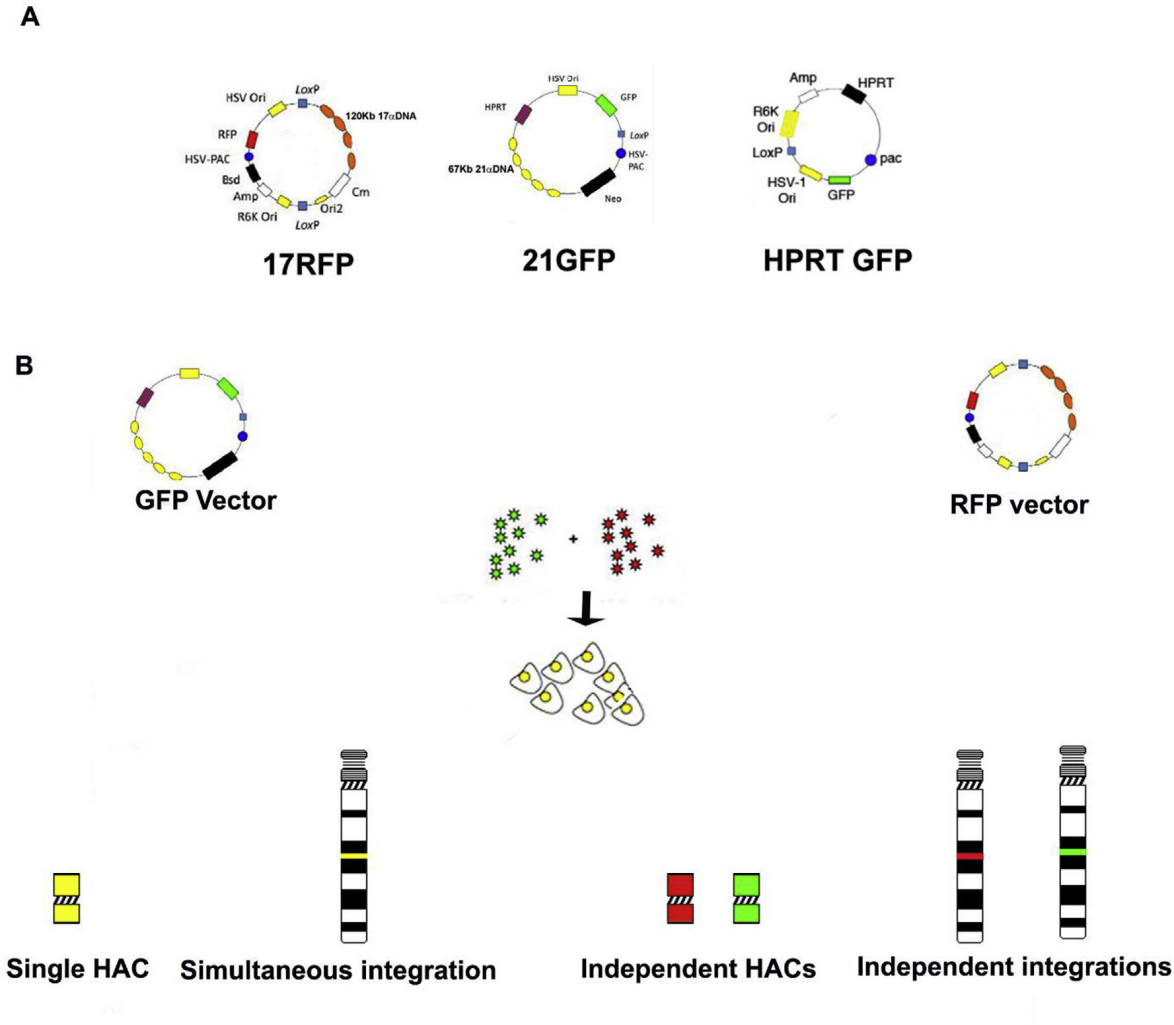
A: Schematic of the three vectors used in this study. The constructs are not drawn to size. B: Diagram of the co-transduction technique, with the possible outcomes of the input DNA.

To determine if the two HAC vectors would form a single functional HAC via HSV-1 amplicon delivery, co-transduction experiments were set up using two different HSV-1 amplicons, one containing the HAC vector with 17α DNA and RFP (17 RFP), and the other amplicon containing HAC vector DNA with 21α DNA and GFP (21 GFP) (Fig. 1). Both HSV-1 HAC amplicons were introduced simultaneously into HT1080 cells, and monitored after 24 h by GFP and RFP fluorescence. In a second experiment, co-transductions were set up with HSV-1 amplicons containing HAC vectors with 17 RFP in one amplicon, and a second HSV-1 amplicon carrying the *HPRT* minigene and GFP as HPRT GFP (Fig. 1A). Both of the amplicons were co-transduced into HT1080, G16-9, human ES (hES) and induced pluripotent stem (iPS) cells.

### 3.1 Generation of a HAC following co-transduction of two HSV-1 amplicons

**Fig. 2:**
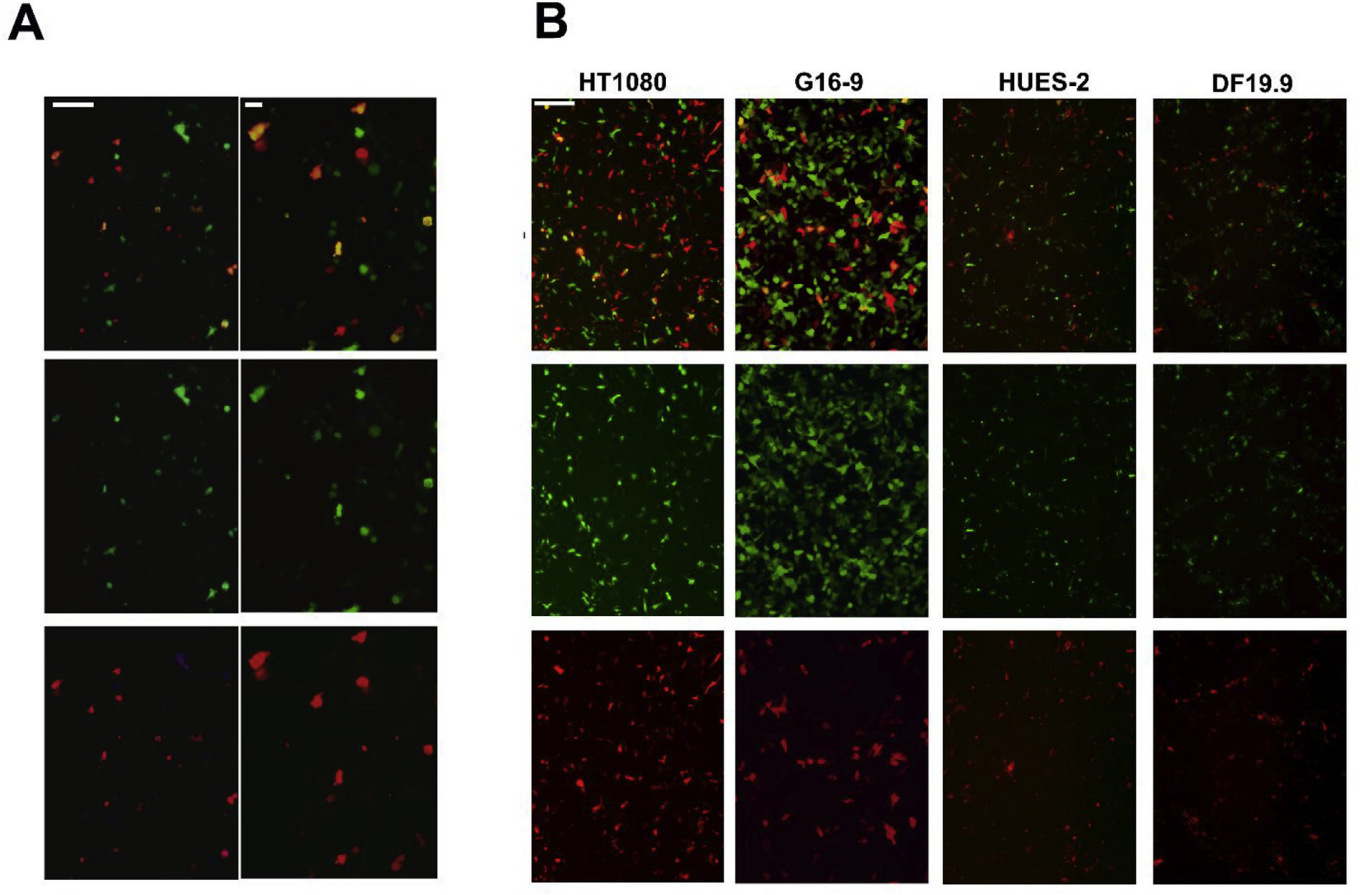
A: 21 GFP+17 RFP in HT0180 24 h after dual transduction. The left panel shows an image acquired with a 4× objective, displaying both GFP and RFP, while the two bottom panels show either GFP or RFP only. The right panel shows the same field, acquired with a 20× objective. The white bar corresponds to 50 μM in both panels. B: 17 RFP and HPRT GFP in different human cell lines 24 h after co-transduction with 17 RFP and HPRT GFP amplicons at a multiplicity of infection of MOI 2. The top panels show both GFP and RFP, while the bottom panels show either GFP or RFP only. The white bar corresponds to 50 μM.

**Fig. 3:**
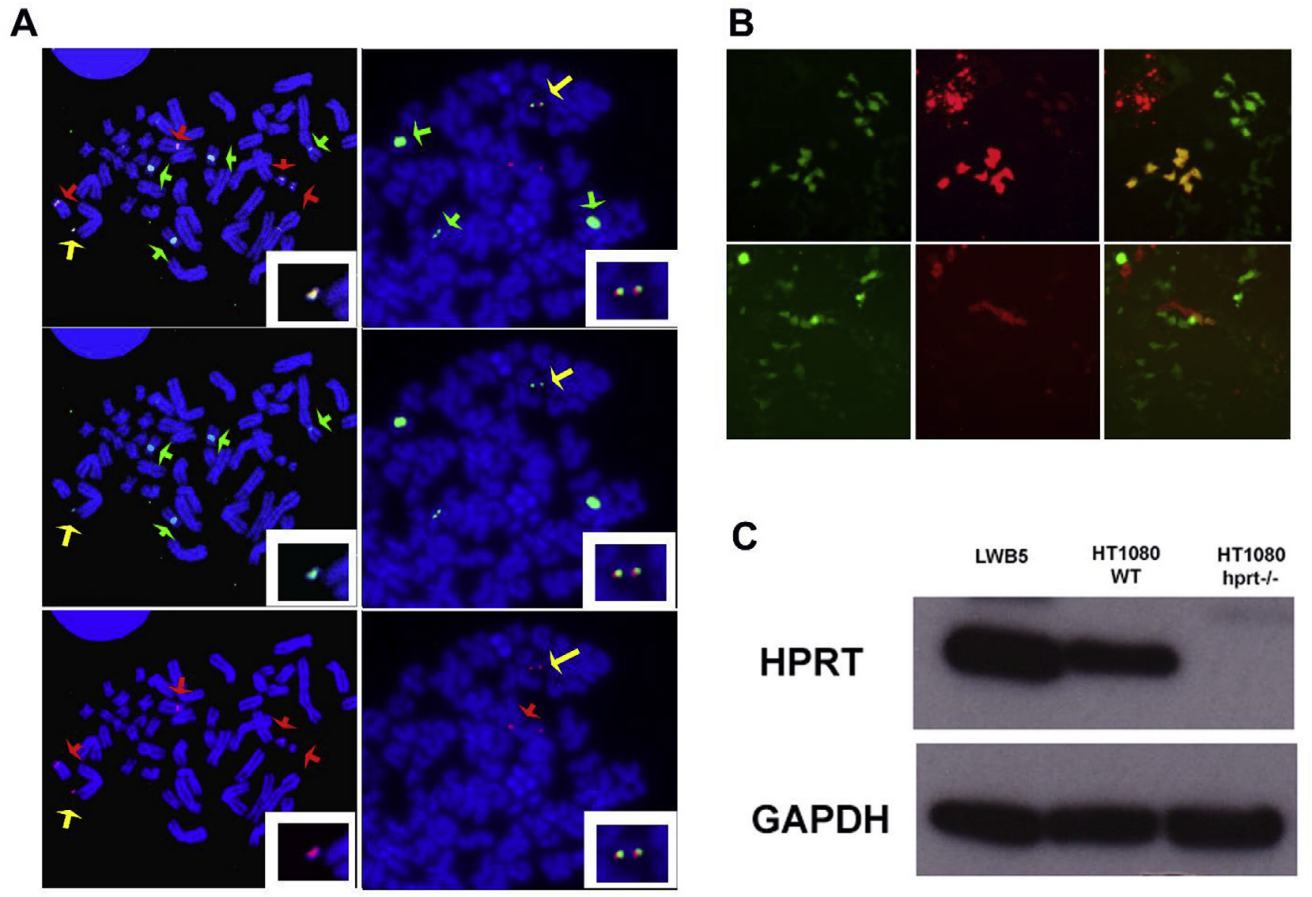
A: Left column D3-3 FISH. The D3-3 metaphase spreads were hybridised with the 17α DNA (green signal) and 21/13α DNA (red signals) probes. The chromosomes were counterstained in DAPI, blue. The HAC (yellow arrow) was labelled by both probes, as visible in the inset. The endogenous chromosome 17 (green arrows) are labelled by 17α only, while the endogenous chromosome 21 and 13 (red arrows) are labelled by the 21/13 probe only. Right column: LWB5 FISH. The LWB5 metaphase spreads were hybridised with the 17α DNA (green signal) and the HPRT DNA (red signal) probes. The chromosomes were counterstained in DAPI, blue. The HAC (yellow arrow) was labelled by both probes, as visible in the inset. The endogenous chromosome 17 (green arrows) were labelled by 17α only, while the endogenous HPRT locus (red arrows) were labelled by the HPRT probe only. B: Clones expressing either GFP or RFP only, or both. Top panels, iPSc DF19.9. Bottom panels, hESc HUES-2. C: Western blot analysis of HPRT expression in the LWB5 clone, and lack of expression in the HT1080 *HPRT*^−/-^ parental cell lines compared to HT10180 WT control. GAPDH was used as loading control.

Following co-transduction of two HSV-1 HAC amplicons, one with 17 RFP and the other with 21 GFP into HT1080 cells at MOI 1 and 5, the cells expressing GFP + RFP were monitored after 24 h and compared to those expressing either GFP or RFP alone (Fig. 2A). The co-transduction efficiencies were determined depending on the MOI titre used. An additional centrifugation step of amplicons at MOI 1 prior to addition to the cells improved the yield (Table 1). In total, 13 positive clones were isolated after selection and 6 were expanded for further analysis. Chromosome metaphase spreads prepared from the 6 clones were analysed by FISH with the 17 and 21 alphoid DNA probes, and immunoFISH with CENP-C. HAC were detected in 2 clones, in up to 40% cells, and integrated DNA was also detected in other cells. Fig. 3A shows the FISH analysis of one clone D3-3, stably carrying the HAC produced from the two inserts, with signal for both the 17α and 21α DNA probes. Positive CENP C staining was also detected on the HAC confirming that a functional centromere had formed [8]. Clone D3.3 was grown in the absence of selection for 30 days to determine the HAC stability. The percentage of HAC containing cells did not change over time, confirming that the HAC possessed and maintained a fully functional centromere.

**Table 1:** Co-transduction efficiency in HT1080 cells after 24 h.

	21 GFP +17 RFP MOI 1		21 GFP +17 RFP MOI 5
	Without centrifugation	With Centrifugation	Without centrifugation
**GFP**	11%	16%	15%
**RFP**	4.0%	16%	13%
**GFP + RFP**	0.4%	6.0%	5.0%

### 3.2 Gene expressing HAC

Two HSV-1 HAC amplicons, 17 RFP and HPRT GFP were co-transduced into human HT1080 (HPRT-), G16-9, hES (HUES 2) and iPS (DF19.9) cells at MO1 2 and appropriate selection applied. The transduced HT1080 (HPRT-) cells were also selected with HAT to detect functional HPRT containing cells. Table 2, indicates the co-transduction efficiency in different cell lines 24 h after transduction, where cells were monitored for expression of GFP + RFP (Fig. 2B). The results showed a high frequency of colocalised RFP + GFP cells in all cell lines transduced, which gave rise to clonal populations showing the expression of both markers (Fig. 3B).

**Table 2:** Efficiency of co-transduction in different human cell lines after 24 h.

	17 RFP	HPRT GFP	17 RFP + HPRT GFP
**HT1080**	7%	11%	3%
**G16**–**9**	18%	26%	3%
**HUES-2**	5%	10%	1%
**iPS DF19**	1%	20%	1%

Further analysis was performed in HT1080 HPRT-cells. Seventeen positive clones were isolated and expanded, and chromosome metaphase spreads prepared from 10 clones. Metaphase spreads were analysed by FISH with the 17α and HPRT DNA probes. Positive HACs with both signals were detected in all cell lines from 20 to 70% of cells, and some cells also contained integrated DNA (Fig. 3A). One clone, LWB5 was analysed by immunoFISH and CENP-C was detected on the HAC (unpublished results). HPRT expression from the LWB5 HAC was analysed by Western blot with an *anti*-HPRT antibody, which detected the presence of HPRT protein in HT1080 HPRT-cells (Fig. 3C). The intensity of the HPRT band in the LWB5 sample indicates that this protein is present in a higher amount compared to the WT HT1080 cells. This was expected since the HPRT gene expression was driven by a strong constitutive promoter. Quantitative RT-PCR data of LWB5 showed expression of the HPRT gene and confirmed that the defect in HT1080 HPRT-cells was complemented.

## 4 Discussion

In this study, we built on previous work where we showed that HSV-1 amplicons containing the vector DNA with sequences for an artificial chromosome including 40 kb of 17 alphoid DNA could generate a functional HAC in human cells including human embryonic stem and neural cells [4]. The results from the present study showed that when two HSV-1 amplicons containing different HAC constructs were introduced into human cells with the aim of generating an artificial chromosome, the exogenous DNAs recombined to generate a single HAC. In initial experiments, two HSV-1 amplicons containing either chromosome 17α or 21α HAC vector DNA were co-transduced into HT1080 cells. Positive clones were identified following selection. One clone D3-3 was analysed in detail by FISH and immunoFISH, and contained a single functional HAC with both 17α and 21α DNA present. The efficiency of co-transduction of the two amplicons relative to the transduction of a single amplicon was comparable in HT1080 cells. In this experiment, MOI 1 following centrifugation of amplicons was found to be more efficient than MOI 5 without centrifugation prior to transduction. In general, the centrifugation of amplicons gives better transduction efficiencies at the MOI used, and we routinely utilise the process prior to transduction.

In a further experiment, we showed that two HSV-1 amplicons, one containing a vector with 40 kb of 17 alphoid DNA and the other with the HPRT gene but lacking alphoid DNA, could form a single HPRT HAC in human HT1080 HPRT-, G16-9, hES and iPS cells. Following selection, we observed co-localisation of both vectors by RFP + GFP fluorescence in all cell lines transduced at a reasonable frequency. At MOI 2 the frequency of co-transduction, in the hES and iPS cells was comparable to that observed in the HT1080 and G16-9 cultured cells although lower than each amplicon transduced singly. In HT1080 HPRT-cells, the FISH and immunoFISH analysis on one of the positive clones identified LWB5, confirmed that a functional HPRT HAC was generated. Analysis of RNA and protein levels in LWB5 indicated that the HPRT gene was expressed and complemented the HPRT deficiency.

## 5 Conclusion

The work presented successfully showed that simultaneous co-infection of two separate HSV-1 amplicons containing different input DNA is an efficient delivery method for studying multigene expression from a HAC generated following co-transduction. The results further show that only one of the HSV-1 amplicons is required to contain 40 kb of alphoid DNA to generate a functional HAC containing the input DNA sequences from both amplicons. The strategy doubles the capacity of the artificial chromosome vectors to accommodate larger stretches of DNA, genes or gene loci within each HSV-1 amplicon, compared to the transfer of a single HSV-1 HAC vector containing amplicon. The results indicate that DNA size will not be a limitation to the HSV-1 amplicon system. Several amplicons containing different genes could be co-transferred simultaneously, and gene expression monitored from the HAC generated within the cells. This study offers a promising approach for studying large genes or multigene expression in a variety of different biochemical systems or complex diseases in human cells, including human embryonic and induced pluripotent cells, and ultimately for gene therapy.

## Declaration of competing interest

The authors declare that they have no conflicts of interest.
